# An overview of the impact of rare disease characteristics on research methodology

**DOI:** 10.1186/s13023-017-0755-5

**Published:** 2018-01-19

**Authors:** Danielle Whicher, Sarah Philbin, Naomi Aronson

**Affiliations:** 1 National Academy of Medicine, 500 5th Street NW, Washington, DC 20001 USA; 2 Patient-Centered Outcomes Research Institute (PCORI), 1919 M Street NW, Washington, DC 20036 USA; 3 Blue Cross Blue Shield Association, 300 E Randolph Street, Chicago, IL 60601 USA

**Keywords:** Rare disease, Research design, Orphan disease, Experimental designs, Registries

## Abstract

**Background:**

About 30 million individuals in the United States are living with a rare disease, which by definition have a prevalence of 200,000 or fewer cases in the United States ([National Organization for Rare Disorders], [About NORD], [2016]). Disease heterogeneity and geographic dispersion add to the difficulty of completing robust studies in small populations. Improving the ability to conduct research on rare diseases would have a significant impact on population health. The purpose of this paper is to raise awareness of methodological approaches that can address the challenges to conducting robust research on rare diseases.

**Approach:**

We conducted a landscape review of available methodological and analytic approaches to address the challenges of rare disease research. Our objectives were to: 1. identify algorithms for matching study design to rare disease attributes and the methodological approaches applicable to these algorithms; 2. draw inferences on how research communities and infrastructure can contribute to the efficiency of research on rare diseases; and 3. to describe methodological approaches in the rare disease portfolio of the Patient-Centered Outcomes Research Institute (PCORI), a funder promoting both rare disease research and research infrastructure.

**Results:**

We identified three algorithms for matching study design to rare disease or intervention characteristics (Gagne, et.al, BMJ 349:g6802, 2014); (Gupta, et.al, J Clin Epidemiol 64:1085-1094, 2011); (Cornu, et. al, Orphet J Rare Dis 8:48,2012) and summarized the applicable methodological and analytic approaches. From this literature we were also able to draw inferences on how an effective research infrastructure can set an agenda, prioritize studies, accelerate accrual, catalyze patient engagement and terminate poorly performing studies. Of the 24 rare disease projects in the PCORI portfolio, 11 are randomized controlled trials (RCTs) using standard designs. Thirteen are observational studies using case-control, prospective cohort, or natural history designs. PCORI has supported the development of 9 Patient-Powered Research Networks (PPRNs) focused on rare diseases.

**Conclusion:**

Matching research design to attributes of rare diseases and interventions can facilitate the completion of RCTs that are adequately powered. An effective research infrastructure can improve efficiency and avoid waste in rare disease research. Our review of the PCORI research portfolio demonstrates that it is feasible to conduct RCTs in rare disease. However, most of these studies are using standard RCT designs. This suggests that use of a broader array of methodological approaches to RCTs --such as adaptive trials, cross-over trials, and early escape designs can improve the productivity of robust research in rare diseases.

## Background

Rare diseases are defined by the Rare Disease Act of 2002 as diseases affecting 200,000 individuals or fewer in the United States [1]. Current estimates indicate that there are close to 7000 rare diseases and that about 30 million individuals in the United States are living with a rare disease [2]. While individually, each rare disease impacts a small population, collectively, a large number of individuals are affected by these conditions. Therefore, improving the ability to conduct research on rare diseases would have a broad population impact.

Research on treatments or management strategies for rare diseases can be challenging primarily due to the limited number of individuals who will be eligible to participate in any given study and uncertainty about or heterogeneity in the natural history of the disease. These smaller populations of eligible individuals can vary in their disease presentation, severity, progression, and exposure to prior treatment and can be geographically dispersed. These issues are not unique to rare diseases but are often magnified for these conditions. The size and characteristics of each affected population impact the type and number of studies that can be conducted due to their influence on factors such as study design, sample size, and power.

To raise awareness among key stakeholders, including researchers, payers, patients, patient advocates, and clinicians, of the available methodological and analytic approaches for addressing these challenges, this paper had three objectives:

1. to identify algorithms for matching study design to rare disease attributes and to summarize the methodological approaches applicable to these algorithms;
2. to draw inferences on how research communities and infrastructure can contribute to the efficiency of research on the treatment and management of rare diseases; and
3. to describe the use of the above approaches in the rare disease portfolio of the Patient-Centered Outcomes Research Institute (PCORI), a funder which is promoting both rare disease research and research infrastructure.

The PCORI is a not-for-profit organization that was created through the Patient Protection and Affordable Care Act of 2010 to fund patient-centered comparative effectiveness research (CER) that provides clinicians, patients, and other stakeholders with the information they need to make informed health care decisions [3]. As part of its charge, PCORI is tasked with supporting CER on rare diseases. PCORI funds both research projects and a national infrastructure to support CER - the National Patient-Centered Clinical Research Network (PCORnet) program. PCORnet is comprised of 13 Clinical Data Research Networks and 20 Patient Powered Research Network. Clinical Data Research Networks are networks that originate in healthcare systems and securely collect health information as part of routine clinical care, whereas Patient Powered Research Networks are networks governed by patients, caregivers, clinicians, researchers, and others focused on a sharing and collecting information particular health care conditions [4].

## Approach

We conducted a landscape review to address the first two objectives, but did not attempt a formal systematic review for practical reasons. First, the purpose of this project was to provide timely information to the PCORI rare disease advisory panel [5]. Second, the potential scope of the literature is vast and our purpose was to get an overview of methodologic approaches rather than to resolve a specific question [6]. The search strategies used to identify relevant articles to address the first two objectives described above are included in the Appendix.

### Objective 1: Algorithms for matching study design to rare disease attributes and main methodological approaches applicable to these algorithms

To identify relevant methodological and analytic approaches to conducting rare disease research, two authors (DW and SP) reviewed the search results and categorized the literature discussing methodological and analytic approaches into three distinct groups: (a) articles that provided a general, high level discussion of different research methods, (b) articles that described the advantages and limits of different methodological and analytic techniques, and (c) articles that discussed the application of research methods in particular clinical settings. The most relevant articles for the purposes of this paper were those within category two that presented algorithms or frameworks designed to help select appropriate study designs for research on rare disease. Other articles were used to generate a list of methodological and analytic approaches used in rare disease research. Where necessary, this literature was supplemented with literature describing the methodological and analytic approaches identified through the literature search.

### Objective 2: Contributions of research communities and infrastructure to the efficiency of research on the treatment and management of rare diseases

To draw inferences on how research communities and infrastructure can contribute to efficiency, the authors first generated examples of how research infrastructure could be leveraged to support rare disease research. The main categories included recruitment and retention, agenda setting, executing a research agenda, terminating poorly performing studies, and executing new studies. Using this list, two authors (DW and SP) then reviewed all articles identified through the literature search, coding those that discussed one or more of the uses of research infrastructure and excluding those that simply described the structure of an existing registry or network.

### Objective 3: Description of approaches in the PCORI rare disease research and infrastructure portfolio

To describe the types of research designs currently being used to support PCORI-funded research, the authors identified currently funded rare disease research projects and extracted the study design and main analytic technique used. We also identified the PCORnet Patient Powered Research Networks that focus on one or more rare diseases and identified the stated purpose of the network or registry.

Several areas that are important to rare disease research were outside the scope of this project. These include pharmacokinetic and pharmacodynamics study designs, guideline development, and the application of evidence-grading methods [7]. Additionally, it was outside the scope of this project to provide descriptions of the study designs. Study design descriptions are widely available in sources on methods both for researchers and interested stakeholders, such as patients, caregivers, clinicians, policy makers, and health system leaders.

## Results

### Objective 1a: Algorithms for matching study design to rare disease attributes

We identified three articles that presented algorithms or structured guidance relating attributes of rare diseases or the interventions of interest to study design decisions. The authors of these three articles generated their recommendations from systematic reviews of the literature [8–10]. Table 1 shows how characteristics of a rare disease, intervention, or outcome may impact study design decisions.

**Table 1:** Features of Rare Diseases, Interventions, or Outcomes Measures that Could Impact Study Design Decisions [8–10]

	Feature of disease, intervention, or outcome measures	Impact on Study Design
Disease Characteristics	Diseases that are life threatening	In placebo controlled RCTs, time on placebo should be minimized
	Diseases in which individuals are often diagnosed when they first have the condition	Prospective inception cohort designs may be useful in establishing temporality among study variables
	Diseases that have an unpredictable disease course	Several experimental designs cannot be used including crossover designs, latin square designs, n-of-1 trials, and randomized withdrawal designs
Intervention Characteristics	Whether the anticipated response to the intervention is non-reversible	Several experimental designs cannot be used including crossover designs, latin square designs, n-of-1 trials, randomized withdrawal designs, early escape, and delayed start designs
	Whether the anticipated response to the intervention is delayed rather than immediate	Several experimental designs cannot be used including crossover designs, latin square designs, n-of-1 trials, early escape designs, and designs that involve adaptive randomization
	Whether the effects on the outcomes are influenced by the order of interventions received*	Several experimental designs cannot be used including crossover designs, latin square designs, and n-of-1 trials
Outcome and prognostic tool characteristics	Whether meaningful surrogate outcomes or composite measures are available or whether statistical techniques for analyzing repeated outcome measures are applicable	In these situations, it may be possible to reduce the sample size needed to answer the study question
	Whether tools are available that can be used to accurately predict prognosis	In these situations, risk-based allocation designs are feasible and it may be possible to reduce the sample size needed if the study focuses on recruiting only patients who are at high risk of progressing. However, enrolling only high risk patients will also reduce the pool of eligible individuals.
	Whether existing research infrastructure exists for the condition of interest, such as a patient registry	In situations where there is existing infrastructure, that infrastructure may be leveraged to recruit eligible participants more rapidly and to implement a study more efficiently
Acceptable levels of uncertainty	Whether decision-makers expected to use the study data are willing accept results from a trial with an alpha >0.05	In these situations, it may be possible to reduce the sample size needed to address the study question are the study would not need to be powered at an alpha ≤0.05

*Unfortunately, this is often not known before a trial has been implemented and trials are often not powered to detect this when it occurs. This can be an important limitation to crossover designs

Gagne and colleagues aimed “to identify innovative approaches to research that have been, or can be, applied to overcome the methodological challenges inherent in the study of rare diseases” [8]. To reduce the required sample size, outcomes that occur more frequently or that occur sooner can be selected. One way to accomplish this is to use composite outcomes or surrogate endpoints. In some cases, using repeated measures in the same individual or using continuous outcome variables may enhance statistical efficiency, depending on the properties of the outcome measures or statistical techniques used [11]. Use of an adaptive trial that allows for pre-specified changes to the study design based on treatment response can improve the efficiency of testing for efficacy by optimizing dosage and delivery without the need for additional trials. Although α ≤ 0.05 is the generally accepted standard for statistical significance in clinical trials, where treatment options are limited, patients may be willing to accept greater uncertainty with consequent reductions in sample size needed to adequately power the study. While we disagree with the proposition that underpowered studies are acceptable because they may ultimately contribute to a meta-analysis, underpowered studies can contribute to a Bayesian model (described below) to inform a future trial that can provide definitive results. Studies in which all participants eventually receive the intervention can attract eligible individuals, potentially improving recruitment and retention.

Where there is an obstacle to randomization due to strong patient preferences, for example a reluctance a placebo or an invasive intervention, the use of an observational design may make recruitment feasible. Potential designs include self-controlled observational study designs (which are similar to crossover designs but do not involve random assignment), case-control designs, and prospective inception cohorts. Propensity scores have been used in an effort to account for confounding due to measured variables. However, Gagne et al. note the inherent limitations of observational methods and state that “greater attention to innovative methods for using observational data to study rare disease health outcomes is needed” [8].

In the second article, Gupta and colleagues summarized a variety of study designs that could be used for studies of rare diseases, including the pros and cons of each design, and developed a framework to help investigators determine when different designs are appropriate [9]. The study designs considered include parallel group designs, crossover designs, n-of-1 trials, adaptive designs, and design combinations. Their framework takes investigators through a series of yes and no questions to assess the usefulness of alternative designs in particular situations. The framework suggests, for example, that crossover and n-of-1 trials should only be used in situations where three conditions are met: the intervention has a predictable and short duration of effect, the disease course is stable over at least two intervention periods, and participants can be retained for at least two intervention periods. However, if the investigators believe it will not be possible to adequately power a crossover trial, than an n-of-1 design should be considered. If the above three conditions are not met and the time between enrollment and outcome assessment is relatively long compared to the time needed to accrue all participants, the framework suggests that investigators should consider a conventional parallel group randomized controlled trial (RCT) design. If the above three conditions are not met and the time between enrollment and outcome assessment is relatively short compared to the time needed to accrue all participants, the framework suggests that investigators should consider adaptive trial designs.

Cornu and colleagues propose a framework where outcomes and responses are key decision points. Investigators are asked to consider first whether the outcomes of interest are reversible or irreversible, then how rapidly individuals are expected to respond to the study intervention(s), followed by whether it is possible to minimize time on placebo, and, finally, whether it is possible to treat all participants enrolled in the study [10]. The algorithm includes twelve possible study designs (parallel group RCT, crossover design, latin square design, n-of-1 trials, randomized placebo phase, stepped wedge, randomized withdrawal, early escape designs, delayed start designs, three stage designs, and adaptive randomization).

In situations where the outcomes included in the trial are reversible and the response to the intervention is relatively quick (within a few weeks), all study designs are possible. If the outcomes are reversible and the response to the intervention is slow, crossover designs including latin square and n-of-1 trials are not feasible, nor are early escape, delayed start, three stage designs, or designs involving adaptive randomization. In situations where outcomes are not reversible but the response to the intervention is relatively fast, crossover designs, including latin square and n-of-1 trials are not feasible, nor are randomized withdrawal, early escape, delayed start, or three stage designs.

If the response to the intervention is relatively slow, in addition to the designs listed above, adaptive randomization is also no longer feasible. The remaining decision nodes ask the investigators to consider the feasibility of minimizing time on placebo and, if it is feasible, whether it is also feasible to implement a design that ensures that all participants will receive active treatment by the end of the study. Similar to Gagne and colleagues, these authors note that designs that can minimize time on placebo and that ensure that all participants will receive a treatment are more attractive to eligible individuals. Study designs that minimize participant time on placebo include delayed start, randomized placebo phase, stepped wedge, randomized withdrawal, early escape, three stage, and adaptive randomization designs. Study designs that also ensure that all participants receive active treatment by the end of the study include delayed start, randomized placebo phase trials, and stepped wedge designs [9].

### Objective 1b. Main methodological approaches

Table 2 is an overview of experimental and non-experimental designs that might be used to address the research challenges posed by rare diseases. Within the literature reviewed, crossover RCTs and adaptive RCTs were discussed most frequently [8, 9, 12–22]. The advantages of a crossover RCT design are that participants are guaranteed to be exposed to the active treatment, enhancing recruitment, and each participant serves as both in the intervention and control group, which may reduce variance and the likelihood of confounding [8]. Because participants serve as their own control, crossover designs require fewer participants when compared to traditional RCTs. The advantages of adaptive RCTs include a reduced number of participants who are recruited to inferior treatment arms and the ability to compare multiple treatment options with constrained sample sizes [8].

**Table 2:** Possible Study Designs for Rare Disease Research

	Design Type	Description
Experimental	Crossover RCT	• Patients are guaranteed to receive active treatment and spend less time on placebo
		• Patients receive two interventions in sequence randomly, with a washout period between interventions
		• Each participant serves as his or her own control, thereby reducing sample size requirements
		• Latin square allows for multiple interventions in randomized sequence
		• Each intervention appears only once in each sequence and intervention period
		• Design variations include N-of-1 trials
	Adaptive RCT	• This design can increase the proportion of patients assigned to the more favorable treatment, which can contribute to a greater number of willing participant
		• Adaptive treatment allocation designs test the null hypothesis in a series of interim analyses; these analyses then influence subsequent randomization in the next phase
		• Bayesian analyses (allowing updates of prior probabilities) or frequentist approaches can be used
		• Adaptive treatment allocation designs allow the probability of being randomized to an intervention to change during the enrollment period; the probability of being randomized will increasingly favor the arm with the more promising results (play the winner) or increasingly penalize the arm with less promising results (drop the loser
		• Adaptive designs can be used to narrow from a selection of doses (ranking and selection designs) rather than rejecting a null hypothesis
		• Adaptive designs can be used to select among subpopulations and thereby balance covariates (covariate-adaptive randomization) and help address underlying heterogeneity
		• Design variations include sequential RCTs and ranking and selection RCTs
	Randomized Placebo Phase	• This design minimizes the length of time that patients are on placebo with all patients receiving treatment in the end. Limited time on placebo is beneficial for conditions with a rapid unfavorable evolution
		• Design variations include stepped wedge, randomized withdrawal, and three-stage trials. In stepped wedged designs, randomization occurs at crossover to different treatment
	Risk-based Allocation	• Low-risk patients are randomized to high-dose and standard treatment, high-risk patients receive high-dosetreatment, thereby addressing concerns about the ethics of withholding treatment from high-risk patients
		• A combined analysis allows the prediction of the added benefit of high-dose treatment
Non-experimental	Case-control study^a^	• This design offers patients with diseases that have long latency periods the opportunity to participate in research
		• Study participants with the disease (cases) are compared to participants without the disease (controls) in an effort to identify factors that may contribute to a particular outcome
		• To address concern that cases and controls may differ in characteristics other than the condition studied, cases and controls can be matched on other characteristics (ex. Age, race, sex)
	Cohorts with historic controls [Natural History Studies]	• This design provides patients with the opportunity to learn about different treatments
		• Comparison of prospectively treated patients with historic controls reduces recruitment burden for the control arm
	Pre-post designs	• Patients receive usual care or standard intervention followed by tested treatment. Patients receive an active treatment throughout the course of the study
		• Requires a detailed understanding of the natural history of the disease to avoid issues of regression to the mean

Descriptions taken from the PCORI Landscape Review on Rare Disease Research Registries unless otherwise noted

^a^Gordis, L. (2009). *Epidemiology* (4th ed.). Philadelphia: Elsevier/Saunders

In addition to the study designs described in Table 2, other experimental designs include repeated measurement designs, factorial designs, and “early escape” in RCT. In a repeated measurement study, multiple observations of response variables are taken from each participant, allowing for within-subject comparisons and increasing the number of data points [23]. In what is essentially a four arm RCT, factorial design involves double randomization in which two comparisons are made concurrently as if conducting two simultaneous studies in the same patient population with the assumption that there is no interaction between the two treatments (i.e. the biologic effect of the first intervention is not mediated or modified by the second intervention) [22]. The benefit of a factorial design is that it allows investigators to answer two research questions within the same trial. Applicable to various trial designs, in “early escape” designs patients can withdraw from the trial either by choice or if they meet a priori criteria listed in the protocol, possibly leading to enhanced retention and power [24]. A prospective inception cohort is another relevant study design of interest but is non-experimental. In this design, cohort inception takes place at the time of medical diagnosis or start of treatment, allowing researchers to establish temporality among study variables, such as baseline confounders and exposures, and to capture outcomes that occur shortly after a participant enters the cohort [8].

The research challenges posed by the characteristics of rare diseases also impact analytic methods. In the literature reviewed on analytic methods in rare disease research, Bayesian analysis was by far the most frequently discussed technique [8, 9, 14–18, 25]. Bayesian analysis provides formal incorporation of prior information or external evidence into the analysis, allowing a greater amount of information to be gained from a smaller number of subjects. A central component of Bayesian analysis is prior probability distribution of the variable of interest from the external data. This distribution is integrated with the distribution of the internal data, yielding the posterior probability distribution [25]. Because of the impact that the prior information has on the analyses, it is important that investigators using Bayesian methods carefully consider the appropriateness of that information before formally incorporating it into the prior distribution or before deciding to use a Bayesian approach [20]. Bayesian methods impact not only the analytic process but also provide a framework that guides the entire research process in both study design and execution.

### Objective 2: Contributions of research communities and infrastructure to the efficiency of research on the treatment and management of rare diseases

Research infrastructure such as registries and research networks might promote the efficiency and success of rare disease research. The literature most frequently discussed the benefits of existing infrastructure, such as contact registries, in order to support recruitment and retention efforts. Several authors describe the establishment of contact registries that store information on individuals who have indicated that they are willing to be contacted about clinical research that they may be eligible for [26–28]. Of these articles that describe research and contact registries, several describe the benefits of having an established infrastructure in terms of facilitating the rapid implementation of studies by expediting enrollment [27–31]. One article described how the research team leveraged their registry to support patient and family engagement in designing research studies [26].

Other potential uses of research infrastructure that were not described in the literature identified through the search but that were identified by the authors of this paper include research prioritization agenda setting, executing a research agenda, and stopping poorly performing studies. A 2010 report by the National Academies of Sciences entitled *Rare Diseases and Orphan Products: Accelerating Research and Development* described the potential benefits of registries in accelerating research for rare disease. However, the report also notes that currently “no uniform, accepted standards govern the collection, organization, or availability of these data. The result is sometimes wasteful duplication and sometimes underuse of information or samples contributed by patients or research participants” [32]. To remedy this issue, the report suggests that there is a need to move toward common standards and a “freely available platform for creating or restructuring patient registries and biorepositories for rare diseases and for sharing de-identified data” [32]. One such initiative that aims to accomplish these objectives is RD-CONNECT. RD-CONNECT was funded in 2012 by the European Union’s Seventh Framework Program under the International Rare Diseases Research Consortium (IRDiRC). Two of the stated goals of this initiative are to develop “an integrated platform to host and analyze genomic and clinical data from research projects” and “common infrastructures and data elements for rare disease patient registries” [33].

### Objective 3: Description of approaches in the PCORI rare disease research and infrastructure portfolio

Tables 3 and 4 are an overview of the rare disease projects within the PCORI portfolio. Table 3 describes funded research on rare diseases as of June 20, 2017, showing both the study design and analytic technique used. The most common study designs used in projects within the PCORI research portfolio are standard RCTs and observational designs. Of the 24 rare disease projects, 11 are RCTs and 13 are observational. One of the RCTs indicated that a Bayesian framework. The observational studies are case-control designs and prospective observational cohorts; 5 are using propensity score analysis. Among both experimental and non-experimental designs, 6 projects are using survival or Kaplan-Meier analysis.

**Table 3:** PCORI Rare Disease Portfolio: Study Designs and Analytic Techniques

Project Title (Condition in Bold)	Study Design	Analytic Technique
Home or Away from Home: Comparing Clinician and Patient/Family-Centered Outcomes Relevant to the Care of Pediatric Acute Myeloid Leukemia during Periods of Neutropenia	Retrospective and prospective observational cohort (Also has a qualitative focus group component)	Propensity score analysis; regression analysis (linear mixed effect models); thematic qualitative analysis
Intervention and Outcomes in Duarte Galactosemia	Case-control observational	Standard Multivariate analysis; generalized estimating equations
Treatment Alternatives in Adult Rare Disease; Assessment of Options in Idiopathic Subglottic Stenosis	Prospective observational cohort	Standard multivariate analysis; survival or Kaplan Meier Analysis
Collaborative Assessment of Pediatric Transverse Myelitis: Understand, Reveal, Educate (CAPTURE) Study	Prospective observational cohort	Propensity scores; sensitivity analysis; mixed effects linear models
The Impact of Self-Management with Probiotics on Urinary Symptoms and the Urine Microbiome in Individuals with Spinal Cord Injury (SCI) and Spina Bifida (SB)	Prospective observational cohort	Linear mixed-effects modeling
Anti-TNF Monotherapy versus Combination Therapy with Low Dose Methotrexate in Pediatric Crohn’s Disease	Randomized, double-blind, multicenter pragmatic clinical trial	Standard multivariate analysis; Survival or Kaplan Meier Analysis
Comparative Effectiveness of CARRA Treatment Strategies for Polyarticular Juvenile Idiopathic Arthritis	prospective observational cohort	Bayesian Analytic Approach, including logistic regression model, sensitivity analysis, linear mixed effects model
Comparative Effectiveness of a Decision Aid for Therapeutic Options in Sickle Cell Disease	RCT; Also descriptive observation study	Specifics not discussed in app. Endpoints for RCT include decisional process and decisional bias
Patient Centered Comprehensive Medication Adherence Management System to Improve Effectiveness of Disease Modifying Therapy with Hydroxyurea in Patients with Sickle Cell Disease	RCT	Linear mixed effects regression models
Comparing patient centered outcomes in the management of pain between emergency departments and dedicated acute care facilities for adults with sickle cell disease	prospective observational cohort	Multilevel regression modelling - Propensity scores
Posterior fossa decompression with or without duraplasty for Chiari type I malformation with syringomyelia.	Cluster-RCT	Plan to use weighted analyses if there is a balance in prognostic factors; Heterogeneity of Treatment Effect (exploratory analysis)
Relapsed childhood neuroblastoma as a model for parental end-of-life decision-making	Prospective longitudinal cohort study (multicenter)/nested case-control study	Standard multivariate analysis; nested case control analysis;
Taking Charge of Systemic Sclerosis: Improving Patient Outcomes Through Self-Management	RCT	Standard univariate and multivariate analysis
Comparative Efficacy of Therapies for Eosinophilic Esophagitis	RCT	Sensitivity analysis; heterogeneity of treatment effect analyses; standard multivariate techniques
Decision Support for Parents Receiving Genetic Information about Child’s Rare Disease	Prospective observational cohort	Thematic Analysis (of interviews, focus groups, surveys)
Individualized Patient Decision Making for Treatment Choices among Minorities with Lupus	RCT	Regression Analysis
Tools and Information to Guide Choice of Therapies in Older & Medically Infirm Patients with Acute Myeloid Leukemia	Prospective observational cohort	Sensitivity Analysis; Propensity Scores; survival-Kaplan modeling; heterogeneity of Treatment Effect
Comparative effectiveness of therapy in rare diseases: Liver transplantation vs. conservative management of urea cycle disorders.	Prospective observational cohort with retrospective components	Propensity scores, survival or Kaplan Meier Analysis
A randomized controlled trial of anterior versus posterior entry site for cerebrospinal fluid shunt insertion (hydrocephalus)	RCT	Survival-Kaplan Model; Regression Analysis
Enhancing Genomic Laboratory Reports to Enhance Communication and Empower Patients (Genetic Diseases)	Randomized, single-blinded pre-post intervention trial with crossover	Regression Analysis
Comparative effectiveness and safety of inhaled corticosteroids and antimicrobial compounds for non-CF bronchiectasis	Retrospective observational cohort	Propensity scores; survival or Kaplan Meier Analysis
A Comparison: High Intense periodic vs. Every week therapy in children with cerebral palsy	RCT	Linear mixed modeling; multivariate linear regression; sensitivity analysis
Developmental Trajectories of Impairments, Health, and Participation of Children with Cerebral Palsy	Prospective longitudinal cohort	Cluster analysis; linear and non-linear mixed effects modelling;
A stakeholder-driven comparative effectiveness study of treatments to prevent coronary artery damage in patients with resistant Kawasaki disease.	RCT	Fisher’s exact test; sensitivity analysis; mixed model repeated measures

*In addition to the studies listed above, PCORI funded 4 methods projects designed to improve the methods for conducting research on rare diseases

**Table 4:** PCORnet Patient Powered Research Networks focusing on Rare Diseases

Project Title	Condition	Project Goal
Collaborative Patient-Centered Rare Epilepsy Network	Rare Epilepsy	Goal of the network is to build patient/caregiver-centered database designed to increase research opportunities for patients and caregivers
ALD Connect	X-linked adrenoleukodystrophy	Inventory and collect information from existing patient registries, advocacy groups and design common elements; create a social network platform that enables communication between patients and researchers
The Vasculitis Patient Powered Research Network	Vasculitis	Directly engages patients in order to explore research questions that matter most to patients – involve patients in study design, increase study eligibility
Phelan-McDermid Syndrome Data Network	Phelan-McDermid Syndrome	Encourage active participation from patient families; develop multiple data feeds to extract and link data from patient cohorts
Empowering Patients and Families for Community-Driven Research: The DuchenneConnect Patient-Report Registry Infrastructure Project	Duchenne and Becker Muscular Dystrophies	Balance robust data collection with reducing burden and increasing benefits for registrants; integrate EHRs; evaluate patient-reported outcome accuracy; improve coding and standardize information exchange
NephCure Kidney Network for Patients with Nephrotic Syndrome	Nephrotic Syndrome	Change nature of NKN from static cross-sectional data to patient-reported outcomes database; establish network governance with active patient participation; collect data that are interoperable across research networks
The Patients, Advocates and Rheumatology Teams Network for Research and Service (PARTNERS) Consortium	Juvenile Rheumatic Disease	Extend current registry; create patient-centered learning health system; patients involved in governance structure
PI Patient Research Connection: PI-CONNECT	Primary Immunodeficiency Diseases	Create venue for researcher and patients to communicate about proposed research; use mobile apps to engage population; integrate existing medical records into the network; identify potential markers for risk stratification
Community Engaged Netowrk for All (CENA)	Alström syndrome; Dyskeratosis congenital; Gaucher disease; Hepatitis; Inflammatory breast cancer; Joubert syndrome; Klinefelter syndrome and associated conditions; Metachromatic leukodystrophy; Pseudoxanthoma elasticum (PXE); Psoriasis	Launch or upgrade online registries; participants determine to whom and for what purpose their information is shared; participant-led governance model

Two of the projects illustrate how infrastructure can support rare disease research. The first study, which compares the outcomes of different entry sites for shunt insertion surgeries to treat hydrocephalus, leveraged the existing Hydrocephalus Clinical Research Network to prioritize the study question, to design the study, and to accelerate enrollment. The second study, which compares a combination therapy to monotherapy anti-TNF in the pediatric Crohn’s Disease, leverages the ImproveCareNow network to recruit participants who are starting anti-TNF treatment.

Table 4 summarizes the focus and goal of each of the 9 rare disease PCORNet patient-powered research networks (PPRNs). The PPRNs currently support 16 studies. The network infrastructure supports both identification of potential participants and patient engagement in the development of research questions and study design. In addition, some PPRNs have undertaken projects to improve methods of data collection. For example, the DuchenneConnect Patient-Report Registry Infrastructure Project has as its goal to reduce the burden of data collection, evaluate the accuracy of patient-reported outcomes, improve coding, and standardize information exchange. The NephCure Kidney Network for Patients with Nephrotic Syndrome aims to improve the interoperability of data collection across networks.

## Discussion

Our landscape review of the literature describes available methodological and analytic approaches that could be used to address the challenges of conducting research in rare diseases, especially the challenge of conducting research with small patient populations. We identified three frameworks that attempted to tie attributes of interventions and rare diseases to specific methodological approaches. As displayed in Table 1, specific features of diseases, interventions, and outcomes have implications for study design choices. In some case, these attributes are limitations and exclude certain designs. In other cases, variations on the standard randomized controlled trial can ameliorate such barriers. For example, as shown in Table 2, both crossover and adaptive RCTs can minimize the time that patients spend on placebo or suboptimal treatments. In any disease, especially those that are serious or life threatening, minimizing exposure to placebo or suboptimal interventions can increase patients’ willingness to be recruited into and be retained in a trial. While the literature we identified focuses predominantly on pharmacologic research, many of the principles that apply to pharmacologic studies also apply to device studies. The exception is that in device studies, blinding of participants and research staff is more difficult and may require, for example, independent outcome assessment.

A central problem of rare disease research is how to avoid conducting underpowered studies. Underpowered studies are “waste in research” [34–36]. While we found limited analysis of the benefits of existing infrastructure in the literature we reviewed, we were able to draw inferences about the benefits of leveraging the capabilities of a research community. A prominent use of networks and registries was to promote contact databases for identifying and recruiting eligible participants. In a well-organized research community, effective research prioritization and agenda setting can reduce waste in research. If a research network or registry engages the specialist community for a particular rare disease, that community can collectively determine which research questions are most critical. For example, the Hydrocephalus Clinical Research Network (hcrn.org) lays out a research agenda on its website and the investigators collaborate to complete research initiatives that fulfill the agenda, thus ensuring that there are not multiple studies competing for the same small patient population. Moreover, defining core outcome sets to be used in registries and studies of rare diseases facilitates aggregation of data over time and comparison across interventions and subpopulations. A corollary role for a research network is to terminate poorly performing studies. If it is clear early on that a study will not be successful, those resources can be redirected to more fruitful avenues. However, while disease registries and the communities that maintain them can be critical to advancing rare disease research, propriety data arrangements can complicate the creation and sustainability of a robust registry.

Our review of the PCORI-funded research portfolio shows 11 RCTs in progress, most using standard RCT approaches, which demonstrates that it is possible to conduct randomized comparative trials in rare diseases. Use of a broader array of methodological approaches could expand the range of diseases feasible to study under PCORI funding. Work underway by the PPRNs shows how a network of engaged patients and researchers might make durable improvements to the research infrastructure, as is demonstrated by the examples we provided of the DuchenneConnect Patient-Report Registry Infrastructure Project and the NephCure Kidney Network for Patients with Nephrotic Syndrome.

Opportunities for methods development that were beyond the scope of this review are designs for studying complex interventions and health system-level interventions in rare diseases. Healthcare delivery and behavioral interventions that seek to improve health care management may improve the quality of care delivered to patients with a variety of rare diseases and may therefore have a cross-cutting impact on patient outcomes. However, these interventions are often complex, involving multiple components, and their success is often dependent on the environment in which they are implemented. Presently, the PCORI Methodology Committee is developing standards on comparative effectiveness research on complex interventions [37].

## Conclusion

Improving the ability to conduct research on rare diseases would have a significant impact on population health. While each rare disease affects a relatively small population, collectively a large number of individuals are affected by these conditions. Disease heterogeneity and geographic dispersion further contribute to the difficulty of completing robust studies in small populations. To raise awareness among key stakeholders of methodological and analytic approaches to these challenges we reviewed algorithms for matching study design to rare disease characteristics and summarized applicable methodological and analytic approaches. Use of these approaches can facilitate the completion of RCTs that are adequately powered. From this literature we were also able to draw inferences on how an effective research infrastructure can set an agenda, prioritize studies, accelerate accrual, catalyze patient engagement, and avoid waste in research. Reviewing the Patient Centered Outcomes Research Institute portfolio of funded studies on rare disease, there were 11 RCTs, most using standard designs. This suggests that use of broader array of methodological approaches to RCTs-- such as adaptive trials, cross-over trials, and early escape designs can improve the productivity of robust research in rare diseases.

## Appendix

**Table 5:** Search Strategy for Identifying Peer Reviewed Literature on Rare Disease Methods and Infrastructure

Search String	Number of Articles	Date Performed
“Rare Diseases”[Mesh] AND (“clinical trials as topic”[Mesh] OR “research design”[Mesh]) AND ((“2003/01/01”[PDAT]: “3000/12/31”[PDAT]) AND “humans”[MeSH Terms] AND English[lang])	191	1/2016
(“method”[ti] OR “methods”[ti] OR “methodology”[ti] OR “methodologies”[ti] OR “design”[ti] OR “designs”[ti]) AND (“rare diseases”[tw] OR “rare disease”[tw] OR “rare disorders”[tw] OR “rare disorder”[tw] OR “rare conditions”[tw] OR “rare condition”[tw] OR “orphan disease”[tw] OR “orphan diseases”[tw]) AND ((“2003/01/01”[PDAT]: “3000/12/31”[PDAT]) AND English[lang])	121	3/2016

**Table 6:** Categories Used to Review Articles Identified Through Search on Rare Disease Methods

Broad Categories	Research Designs within each category
Randomized Designs	Parallel-group RCT; Crossover RCT; N-of-1 Trials; Ranking and Selection RCT; Sequential RCT; Adaptive RCT; Internal Pilot; Randomized Placebo Phase; Stepped Wedge; “Early Escape” in RCT; Randomized Withdrawal; Three-stage trial; Boundaries Design; Factorial Design; Phase II Multicenter Open Label; Phase III: Intent to Treat - Randomized, double-blind, Placebo Controlled; RCT with patients grouped by etiology; Phase I/II Proof of Principle; Repeated measurement designs; Single multi-arm trial where a series of research arms are assessed in parallel against a common control
Nonrandomized Controlled Trials	Risk-based allocation; Delayed Start
Observational Designs	Prospective inception cohort; case-control studies; cohorts with historic controls/ Natural History Studies; Pre-post designs; Case reports/ case series
Analytic Methods	Bayesian Analysis; Propensity Scores; Instrumental Variables
Other	Meta-Analysis
